# Acceptance of Surgical and Non-surgical Cosmetic Procedures: A Cross-Sectional Study From Jazan, Saudi Arabia

**DOI:** 10.7759/cureus.54035

**Published:** 2024-02-11

**Authors:** Anas A Sayegh, Ayman M Albarrati, Alhassan H Hobani, Ali M Shawish, Fatmah O Alshekh, Mohammed E Mojiri, Lulwah S Alhumaidan, Rami G Ahmad

**Affiliations:** 1 Department of Surgery, Jazan University, Jazan, SAU; 2 Faculty of Medicine, Jazan University, Jazan, SAU; 3 Unaizah College of Medicine and Medical Sciences, Qassim University, Unaizah, SAU; 4 Psychiatry Section, Department of Medicine, Ministry of National Guard - Health Affairs, Jeddah, SAU; 5 Research Office, King Abdullah International Medical Research Center, Jeddah, SAU

**Keywords:** plastic surgery, acceptance, procedures, surgeries, cosmetic

## Abstract

Background and objective

Cosmetic surgery is a field that primarily focuses on the preservation, rebuilding, or improvement of the physical appearance of an individual through surgical and therapeutic methods. This specialization encompasses various interventions, both surgical, such as blepharoplasty, rhinoplasty, and breast augmentation, and non-surgical, including procedures such as chemical peeling, Botox injections, and dermal fillers. This study aims to assess the acceptance of cosmetic surgeries and non-surgical cosmetic procedures and the reasons for non-acceptance in a population from Jazan, Saudi Arabia.

Methods

This cross-sectional survey study was conducted in the general population of Jazan, Saudi Arabia, between July and August 2023. An online self-administered questionnaire was created using Google Forms and distributed through social media. The acceptance was measured using the Arabic translation of the Acceptance of Cosmetic Surgery Scale (ACSS).

Results

The mean cosmetic surgery acceptance score was 62.1 ± 25.9, whereas the mean non-surgical procedure acceptance score was 63.7 ± 24.5. Engaged and widowed participants had a higher mean acceptance score for cosmetic surgery, whereas divorced participants had a higher mean acceptance score for non-surgical cosmetic procedures. Higher age was associated with higher acceptance of cosmetic surgery (95% CI: 1-15), while having higher income was associated with lower acceptance (95% CI: -14 to -0.32). A higher level of parental education was associated with lower acceptance of surgical and non-surgical cosmetic procedures (95% CI: -23 to -3.5). The perceived lack of a need for cosmetic procedures was the most commonly cited reason for not accepting these procedures, while religious beliefs were the second most common reason.

Conclusion

Non-surgical cosmetic procedures generally had higher acceptance than cosmetic surgeries. Age, sex, marital status, income level, familial influence, and prior experience all played significant roles in shaping these attitudes. The perceived lack of a need for the procedures and religious beliefs were common reasons for not accepting cosmetic procedures.

## Introduction

Cosmetic surgery, a distinct subfield within the realm of medicine, primarily focuses on enhancing an individual’s physical appearance in alignment with specific aesthetic standards while ensuring that the areas being treated maintain satisfactory function [1]. This specialization encompasses various interventions, both surgical (e.g., blepharoplasty, rhinoplasty, and breast augmentation) and non-surgical, including procedures such as chemical peeling, Botox injections, and dermal fillers [2]. The discourse surrounding beauty ideals has led to a significant surge in the prevalence, accessibility, and availability of both surgical and non-surgical cosmetic procedures. According to the International Society of Aesthetic Plastic Surgery (ISAPS), there was a notable 19.3% increase in the number of procedures performed by plastic surgeons worldwide in 2021, and this amounted to more than 12.8 million surgical procedures and 17.5 million non-surgical procedures annually [3].

Traditionally, the scalpel has played a dominant role in the field of cosmetology. However, contemporary approaches utilizing techniques such as lasers, needles, and chemical agents are posing an increasing challenge to surgical incision approaches with scalpels [4]. These non-invasive procedures typically focus on enhancing facial features, improving skin conditions, and addressing hair-related issues. Their proliferation has blurred the boundaries between medical and cosmetic practices, and they have been frequently supplanting more invasive interventions [5]. In fact, in recent years, there has been a decline in the number of patients opting for cosmetic invasive surgery, but the popularity of non-invasive alternatives continues to rise. This shifting trend is influenced by a combination of factors, including epidemiological considerations, the influence of social networks, and individual psychological attributes such as body image, self-esteem, personality traits, and psychopathological conditions [6]. These psychological factors can either positively or negatively impact an individual’s motivation to pursue and undergo cosmetic procedures.

Saudi Arabia, in particular, has witnessed the emergence of cosmetic procedures as a growing phenomenon, and a significant number of both surgical and non-surgical interventions have been reported annually. A study conducted by the International Society of Aesthetic Plastic Surgeons ranked Saudi Arabia 22nd among the top 25 countries with the highest number of cosmetic procedures among women [7]. In a country where lifestyle is deeply influenced by religion, Saudi Arabia has experienced a notable increase in interest in cosmetic procedures in recent years. Despite this, a dearth of reliable information and certain religious interpretations have impeded the growth of cosmetic surgery in the region [8]. However, there is not enough data to shed light on the related trends in the country. Having such data would help identify parts of the population that are likely to opt for such procedures and spread awareness about the risk of cosmetic procedures in these demographics. Given the heightened global popularity of cosmetic interventions and the scarcity of recent data specific to Saudi Arabia, this survey-based study aims to assess the acceptance of cosmetic surgeries and procedures, as well as the reasons for non-acceptance of such procedures, in a population from Jazan in Saudi Arabia.

## Materials and methods

Study setting and population

This descriptive cross-sectional study was conducted in Jazan, one of the 13 regions of Saudi Arabia, which lies in the southwest corner directly north of the border with Yemen. It covers an area of 11,671 km^2^ and includes around 5,000 villages and towns with a total population of 1.7 million. This study used a convenient sample. All Jazan populations were eligible for this study, and only those who responded to the questionnaire, aged 18 and above, and all nationalities (Saudi and non-Saudi) were recruited. People who were aged less than 18, non-Jazan citizens, and those who refused to participate in the study were excluded. The sample size was calculated using the Raosoft sample size calculator [9], and 385 participants were needed to reach a 95% confidence interval and 5% margin of error. Our sample size was 386 participants.

Content of the questionnaire 

An online self-administered questionnaire was created using Google Forms, and the link was distributed through social media platforms, including WhatsApp, Telegram, and X (formerly Twitter). We targeted already existing social groups and accounts that included residents of Jazan and added a request that the questionnaire be shared in WhatsApp groups with family and friends. To avoid duplication of responses, we also added a note to the messages sent on social groups requesting recipients to ignore the message if they had already filled in the questionnaire.

The questionnaire included four sections. The first section focused on sociodemographic characteristics, such as age, sex, marital status, income, parents’ education, the presence of a health practitioner in the family, and history of cosmetic surgery or procedures. The second section included the Acceptance of Cosmetic Surgery Scale (ACSS), a validated scale developed by Henderson-King et al. [10] for the measurement of acceptance of cosmetic surgery. This study uses the Arabic version of the ACSS that was validated by Morait et al. [11] in a Saudi general population and was found to have a Cronbach alpha value of 0.912. Here, we used the ACSS twice - for evaluating acceptance of cosmetic surgeries and for evaluating acceptance of non-surgical cosmetic procedures. It is a 15-item questionnaire in which the questions are scored on a 7-point scale (1 = strongly disagree, 7 = strongly agree), with higher scores indicative of greater endorsement of cosmetic surgery. The ACSS comprises three subscales with five items each, and each of the scales focuses on different reasons for undergoing cosmetic surgery: the intrapersonal subscale (items 1, 2, 4, 5, and 14) assesses self-benefits as a potential reason; the social subscale (items 9, 11, 12, 13, and 15) assesses social reasons; and the consider subscale (items 3, 6, 7, 8, and 10) examines general reasons under various scenarios. For item 10, the score was reversed.

The third section of the questionnaire contains multiple-choice questions on the perceived reasons for not accepting cosmetic surgeries and non-surgical cosmetic procedures. The fourth section assesses the sources of information regarding cosmetic surgeries and non-surgical cosmetic procedures and planned cosmetic surgeries or procedures for the future by asking multiple-choice questions.

Statistical analysis 

The data were cleaned in Excel and imported to R software (version 4.2.2; R Development Core Team, Vienna, Austria). The normality of data distribution was tested using histograms and the Kolmogorov-Smirnov test. Descriptive statistics were used for calculating the mean and standard deviation for the continuous variables and frequencies with percentages for the categorical variables. One-way analysis of variance (ANOVA) and two-sample t-tests were used to identify variables associated with acceptance of cosmetic surgeries and procedures. A multiple linear regression analysis was performed to identify the predictors of acceptance of cosmetic surgeries and procedures. A p-value of ≤ 0.05 was set as the significance level of the study.

Ethical considerations

Ethical approval was secured from the Research Ethics Committee at Jazan University (reference approval no: -44/11/703). The objectives of the study were comprehensively explained to all the participants at the beginning of the questionnaire who were also informed that they could withdraw from the study at any point and assured of the anonymity and confidentiality of their data. The written informed consent of all the participants confirming their voluntary participation was obtained.

## Results

A total of 386 individuals participated in this study. They were divided into three age groups: 18-34 years, 35-54 years, and 55 years and above. The majority were aged 18-34 years (264, 68.39%), and the majority were females (268, 69.43%), for the reason that females are more attracted to cosmetic procedures. Moreover, the majority had a monthly income of more than 10,000 Saudi Riyals (200, 51.81%) (Table 1). With regard to other predominant characteristics of the population, the majority were students (199, 51.55%) and were single (232, 60.10%). In addition, the majority lived in houses that were self-owned or owned by their family (299, 77.46%), and the majority had a health practitioner in the family (257, 66.58%). There were no predominant trends in terms of parental education.

**Table 1:** Sociodemographic characteristics of the study participants (n = 386)

Characteristics	n	%
Age (years)		
18–34	264	68.39%
35–54	111	28.76%
55 and above	11	2.85%
Sex		
Female	268	69.43%
Male	118	30.57%
Nationality		
Non-Saudi	17	4.40%
Saudi	369	95.60%
Marital status		
Single	232	60.10%
Engaged	23	5.96%
Married	105	27.20%
Divorced	19	4.92%
Widowed	7	1.81%
Career status		
Government sector employee	91	23.58%
Private sector employee	30	7.77%
Students	199	51.55%
Self-employed	11	2.85%
Unemployed	44	11.40%
Retired	11	2.85%
Housing type		
Owned house	299	77.46%
Rental	87	22.54%
Monthly family income		
Less than 5,000 Saudi Riyals	81	20.98%
5000–10,000 Saudi Riyals	105	27.20%
More than 10,000 Saudi Riyals	200	51.81%
Do you have a health practitioner in your family?		
No	129	33.42%
Yes	257	66.58%
Father’s level of education		
Doesn’t read or write	31	8.03%
Only he reads and writes	30	7.77%
Elementary school or middle school	49	12.69%
High school	62	16.06%
Bachelor’s degree	40	10.36%
Diploma	144	37.31%
Master’s degree or PhD	30	7.77%
Mother’s level of education		
Doesn’t read or write	75	19.43%
Only she reads and writes	34	8.81%
Elementary school or middle school	39	10.10%
High school	39	10.10%
Bachelor’s degree	39	10.10%
Diploma	130	33.68%
Master’s degree or PhD	30	7.77%

The interpersonal subscale shows the most significance among other subscales with a high total mean of 23.6 ± 8.3, followed by the consider and social subscales, respectively. However, most of the participants strongly agreed with all five questions of the interpersonal subscale. Furthermore, in the consider subscale, the highest mean (4.5 ± 2.3) was for the question "If I knew there would be no negative side effects or pain, I would like to try cosmetic surgery" (Table 2).

**Table 2:** Percentage description of the trend of the answers to the ACSS cosmetic surgery M - mean, SD - standard deviation

Variables	Strongly Disagree	Disagree Somewhat	Disagree a Little	Neutral	Agree a Little	Agree Somewhat	Strongly Agree	M ± SD
Interpersonal								
It makes sense to have cosmetic surgery rather than spending years feeling bad about the way you look.	39 (10.1%)	30 (7.8%)	25 (6.5%)	66 (17.1%)	47 (12.2%)	68 (17.6%)	111 (28.8%)	4.8±1.9
Cosmetic surgery is a good thing because it can help people feel better about themselves.	18 (4.7%)	7 (1.8%)	20 (5.2%)	73 (18.9%)	64 (16.6%)	86 (22.3%)	118 (30.6%)	5.3±1.7
People who are very unhappy with their physical appearance should consider cosmetic surgery as one option.	64 (16.6%)	26 (6.7%)	43 (11.1%)	58 (15%)	48 (12.4%)	54 (14%)	93 (24.1%)	4.3±2.2
If cosmetic surgery can make someone happier with the way they look, then they should try it.	74 (19.2%)	27 (7%)	37 (9.6%)	54 (14%)	47 (12.2%)	54 (14%)	93 (24.1%)	4.3±2.2
Cosmetic surgery can be a big benefit to people’s self‐image.	34 (8.8%)	14 (3.6%)	15 (3.9%)	87 (22.5%)	75 (19.4%)	66 (17.1%)	95 (24.6%)	4.9±1.8
Total								23.6±8.3
Social								
I would seriously consider having cosmetic surgery if my partner thought it was a good idea.	144 (37.3%)	19 (4.9%)	32 (8.3%)	33 (8.5%)	39 (10.1%)	43 (11.1%)	76 (19.7%)	3.6±2.4
I would think about having cosmetic surgery to keep looking young.	124 (32.1%)	27 (7%)	33 (8.5%)	57 (14.8%)	31 (8%)	43 (11.1%)	71 (18.4%)	3.7±2.3
If it would benefit my career, I would think about having plastic surgery.	90 (23.3%)	26 (6.7%)	25 (6.5%)	56 (14.5%)	40 (10.4%)	52 (13.5%)	97 (25.1%)	4.2±2.3
Would seriously consider having cosmetic surgery, if I thought my partner would find me more attractive.	133 (34.5%)	27 (7%)	29 (7.5%)	49 (12.7%)	34 (8.8%)	30 (7.8%)	84 (21.8%)	3.6±2.3
If a cosmetic surgery procedure would make me more attractive to others, I would think about trying it	108 (28%)	31 (8%)	43 (11.1%)	53 (13.7%)	32 (8.3%)	44 (11.4%)	75 (19.4%)	3.8±2.3
Total								18.9±10.3
Considered								
In the future, I could end up having some kind of cosmetic surgery	86 (22.3%)	28 (7.3%)	22 (5.7%)	80 (20.7%)	51 (13.2%)	46 (11.9%)	73 (18.9%)	4.1±2.1
If I could have cosmetic surgery done for free, I would consider trying it	102 (26.4%)	26 (6.7%)	33 (8.5%)	40 (10.4%)	34 (8.8%)	43 (11.1%)	108 (28%)	4.1±2.4
If I knew there would be no negative side effects or pain, I would like to try cosmetic surgery.	81 (21%)	22 (5.7%)	28 (7.3%)	41 (10.6%)	46 (11.9%)	47 (12.2%)	121 (31.3%)	4.5±2.3
I have sometimes thought about having cosmetic surgery.	115 (29.8%)	22 (5.7%)	23 (6%)	38 (9.8%)	61 (15.8%)	46 (11.9%)	81 (21%)	3.9±2.3
I would never have any kind of plastic surgery.	26 (6.7%)	20 (5.2%)	41 (10.6%)	76 (19.7%)	26 (6.7%)	52 (13.5%)	145 (37.6%)	3.0±2.0
Total								19.6±9.3

The interpersonal subscale shows the most significance among other subscales with a high total mean of 23.2 ± 8.3, followed by the consider and social subscales, respectively. Additionally, the consider subscale shows a high mean in three questions: "In the future, I could end up having some kind of non-surgical procedures such as filer, botox & laser", "If I knew there would be no negative side effects or pain, I would like to try non-surgical procedures such as filer, botox & laser", and "I have sometimes thought about having non-surgical procedures such as filer, botox & laser" (Table 3).

**Table 3:** Percentage description of the trend of the answers to the ACSS non-surgical cosmetic procedure M - mean, SD - standard deviation

Variables	Strongly Disagree	Disagree Somewhat	Disagree a Little	Neutral	Agree a Little	Agree Somewhat	Strongly Agree	M ± SD
Interpersonal								
It makes sense to have non-surgical procedures such as filer, botox & laser rather than spending years feeling bad about the way you look.	48 (12.4%)	19 (4.9%)	28 (7.3%)	82 (21.2%)	65 (16.8%)	57 (14.8%)	87 (22.5%)	4.6±1.9
Non-surgical procedures such as filer, botox & laser is a good thing because it can help people feel better about themselves.	37 (9.6%)	12 (3.1%)	12 (3.1%)	87 (22.5%)	81 (21%)	70 (18.1%)	87 (22.5%)	4.9±1.8
People who are very unhappy with their physical appearance should consider Non-surgical procedures such as filer, botox & laser as one option.	45 (11.7%)	20 (5.2%)	32 (8.3%)	80 (20.7%)	67 (17.4%)	64 (16.6%)	78 (20.2%)	4.5±1.9
If non-surgical procedures such as filer, botox & laser can make someone happier with the way they look, then they should try it.	56 (14.5%)	19 (4.9%)	35 (9.1%)	78 (20.2%)	54 (14%)	60 (15.5%)	84 (21.8%)	4.4±2.1
Non-surgical procedures such as filer, botox & laser can be a big benefit to people’s self‐image.	38 (9.8%)	14 (3.6%)	17 (4.4%)	86 (22.3%)	75 (19.4%)	65 (16.8%)	91 (23.6%)	4.8±1.9
Total								23.2±8.3
Social								
I would seriously consider having non-surgical procedures such as filer, botox & laser, if my partner thought it was a good idea.	108 (28%)	25 (6.5%)	31 (8%)	71 (18.4%)	36 (9.3%)	37 (9.6%)	78 (20.2%)	3.8±2.3
I would think about having non-surgical procedures such as filer, botox & laser to keep looking young.	96 (24.9%)	30 (7.8%)	35 (9.1%)	49 (12.7%)	54 (14%)	40 (10.4%)	82 (21.2%)	4±2.3
If it would benefit my career, I would think about having Non-surgical procedures such as filer, botox & laser.	85 (22%)	27 (7%)	24 (6.2%)	65 (16.8%)	52 (13.5%)	31 (8%)	102 (26.4%)	4.2±2.3
would seriously consider having Non-surgical procedures such as filer, botox & laser, if I thought my partner would find me more attractive.	106 (27.5%)	30 (7.8%)	22 (5.7%)	61 (15.8%)	42 (10.9%)	35 (9.1%)	90 (23.3%)	4±2.3
If a non-surgical procedure such as filer, botox & laser would make me more attractive to others, I would think about trying it.	91 (23.6%)	33 (8.5%)	32 (8.3%)	70 (18.1%)	49 (12.7%)	31 (8%)	80 (20.7%)	4±2.2
Total								19.9±10.1
Considered								
In the future, I could end up having some kind of Non-surgical procedures such as filer, botox & laser.	74 (19.2%)	26 (6.7%)	25 (6.5%)	69 (17.9%)	60 (15.5%)	44 (11.4%)	88 (22.8%)	4.3±2.2
If I could have non-surgical procedures such as filer, botox & laser done for free, I would consider trying it.	79 (20.5%)	32 (8.3%)	24 (6.2%)	67 (17.4%)	47 (12.2%)	48 (12.4%)	89 (23.1%)	4.2±2.2
If I knew there would be no negative side effects or pain, I would like to try Non-surgical procedures such as filer, botox & laser.	68 (17.6%)	32 (8.3%)	24 (6.2%)	69 (17.9%)	50 (13%)	51 (13.2%)	92 (23.8%)	4.4±2.1
I have sometimes thought about having non-surgical procedures such as filer, botox & laser.	78 (20.2%)	20 (5.2%)	26 (6.7%)	64 (16.6%)	63 (16.3%)	42 (10.9%)	93 (24.1%)	4.3±2.2
I would never have any kind of Non-surgical procedures such as filer, botox & laser.	32 (8.3%)	22 (5.7%)	40 (10.4%)	104 (26.9%)	35 (9.1%)	40 (10.4%)	113 (29.3%)	3.3±2.0
Total								20.5±8.3

The mean total score for acceptance of cosmetic surgery was 62.1 ± 25.9. Overall, for all demographic and other categories, the interpersonal subscale scores were higher than the scores for the other two subscales, with one exception in the widowed group, which had higher scores on the social subscale

With regard to age-dependent differences, an increase in age was significantly associated with higher scores for the three subscales and a higher overall score (p < 0.001); in contrast, no sex-dependent differences were observed. With regard to marital status, engaged and widowed participants had higher overall scores than married, divorced, and single participants (p < 0.001). Earning less than 5,000 Saudi Riyals per month was associated with higher mean scores for acceptance of cosmetic surgery (p < 0.05). Parents' level of education was also significantly associated with acceptance of cosmetic surgery, as individuals with parents who were illiterate had higher mean scores for acceptance of cosmetic surgery (p < 0.001). However, having a health practitioner in the family did not have a significant effect on the overall acceptance score. Having a history of cosmetic surgery was also associated with higher mean scores of acceptance (p < 0.001). Finally, being aware of the risks of cosmetic surgery was associated with lower scores on the interpersonal subscale (p = 0.022) but did not affect the scores for the other two subscales.

Briefly, perceived self-benefits were generally associated with higher acceptance scores than perceived social and other benefits. With regard to the influential factors, age, marital status, monthly income, parents’ education level, a history of cosmetic surgery, and awareness of the risk of cosmetic surgery were found to significantly affect the acceptance scores (Table 4).

**Table 4:** Total ACSS scores and subscale scores for cosmetic surgery according to demographic and other variables M - mean, SD - standard deviation; p < 0.05 (significant)

Mean score	Intrapersonal scale (M ± SD: 23.6 ± 8.3)		Social scale (M ± SD: 18.9 ± 10.3)		Consider scale (M ± SD: 19.6 ± 9.3)		Overall score (M ± SD: 62.1 ± 25.9)	
Characteristic	N = 386	p-value^1^	N = 386	p-value^1^	N = 386	p-value^1^	N = 386	p-value^1^
Age (years)		<0.001		<0.001		<0.001		<0.001
18–34	22 ± 8		17 ± 10		18 ± 9		57 ± 25	
35–54	27 ± 8		23 ± 10		23 ± 9		73 ± 26	
55 and above	29 ± 8		23 ± 10		23 ± 8		75 ± 24	
Sex		0.7		0.9		0.4		0.7
Female	24 ± 8		19 ± 11		20 ± 9		62 ± 26	
Male	23 ± 8		19 ± 10		19 ± 9		61 ± 26	
Marital status		<0.001		<0.001		<0.001		<0.001
Single	22 ± 8		16 ± 9		18 ± 9		56 ± 24	
Engaged	28 ± 7		25 ± 10		23 ± 8		76 ± 23	
Married	26 ± 9		21 ± 11		22 ± 9		69 ± 27	
Divorced	26 ± 10		25 ± 11		23 ± 9		74 ± 27	
Widowed	26 ± 8		27 ± 8		24 ± 6		76 ± 21	
Monthly income		0.015		<0.001		0.002		<0.001
Less than 5,000 Saudi Riyals	26 ± 8		23 ± 10		23 ± 9		72 ± 24	
5000–10,000 Saudi Riyals	23 ± 9		19 ± 11		20 ± 9		62 ± 27	
More than 10,000 Saudi Riyals	23 ± 8		17 ± 10		18 ± 9		58 ± 25	
Do you have a health practitioner in your family?		0.12		0.2		0.3		0.15
No	23 ± 9		18 ± 11		19 ± 10		59 ± 27	
Yes	24 ± 8		19 ± 10		20 ± 9		63 ± 26	
Father’s level of education		<0.001		<0.001		<0.001		<0.001
Doesn’t read or write	29 ± 6		27 ± 10		26 ± 8		82 ± 21	
Only he reads and writes	27 ± 7		24 ± 10		22 ± 9		73 ± 25	
Elementary school or middle school	24 ± 9		20 ± 11		20 ± 9		64 ± 27	
High school	23 ± 8		19 ± 11		20 ± 9		61 ± 27	
Bachelor’s degree	24 ± 8		16 ± 9		19 ± 8		58 ± 22	
Diploma	22 ± 8		16 ± 9		18 ± 9		55 ± 25	
Master’s degree or PhD	24 ± 8		20 ± 9		21 ± 10		66 ± 25	
Mother’s level of education		0.002		<0.001		0.002		<0.001
Doesn’t read or write	27 ± 8		23 ± 11		23 ± 9		73 ± 27	
Only she reads and writes	24 ± 8		21 ± 10		22 ± 8		67 ± 24	
Elementary school or middle school	22 ± 9		18 ± 10		17 ± 9		58 ± 28	
High school	21 ± 8		15 ± 10		16 ± 9		52 ± 25	
Bachelor’s degree	22 ± 7		17 ± 9		19 ± 8		59 ± 22	
Diploma	23 ± 8		18 ± 10		20 ± 9		61 ± 25	
Master’s degree or PhD	23 ± 9		16 ± 11		17 ± 10		56 ± 27	
Have you ever undergone cosmetic surgery?		<0.001		<0.001		<0.001		<0.001
No	23 ± 8		18 ± 10		19 ± 9		59 ± 26	
Yes	31 ± 4		29 ± 7		27 ± 5		87 ± 13	
Are you aware of the risks of cosmetic surgery?		0.022		0.11		0.14		0.060
No	25 ± 8		20 ± 10		21 ± 9		66 ± 26	
Yes	23 ± 8		18 ± 10		19 ± 9		61 ± 26	
^1^One-way ANOVA; Welch two-sample t-test								

The mean total score for acceptance of non-surgical cosmetic procedures was 63.7 ± 24.5. As observed for acceptance of cosmetic surgeries, overall, the scores for the interpersonal subscale were higher than the scores for the other two subscales, with the exception of widowed and engaged individuals, who scored more on the social subscale than on the other two subscales. With regard to age, as observed for cosmetic surgery, an increase in age was significantly associated with higher mean acceptance scores for non-surgical cosmetic procedures in the three subscales and the overall scale. In contrast to the observation for cosmetic surgery, a sex-based trend was observed for non-surgical procedures: females had higher overall acceptance scores (p = 0.015), as well as higher scores on the intrapersonal (p = 0.017) and consider subscales (p < 0.001). Divorced participants had significantly higher overall scores and subscale scores than married, single, engaged, and widowed participants (p < 0.001). Further, a monthly income of less than 5000 Saudi Riyals was significantly associated with higher overall and social subscale scores (p = 0.009 and p < 0.001, respectively). Having a health practitioner in the family was associated with higher intrapersonal subscale scores (p = 0.024) and overall scale scores (p = 0.032), even though this factor was not associated with acceptance of cosmetic surgery. Lower parental education levels were associated with higher acceptance scores on all the subscales, as observed for acceptance of cosmetic surgery. Finally, having a history of non-surgical cosmetic procedures was associated with higher acceptance scores, but being aware of the risks did not seem to affect acceptance.

Briefly, the scores for the interpersonal subscale were generally higher than those for the other two subscales, as observed for acceptance of cosmetic surgeries. Further, the influential factors were age, sex, marital status, monthly income, parental education levels, having a health practitioner in the family, and having a history of cosmetic procedures (Table 5). Most of these factors were also found to influence acceptance of cosmetic surgery, but some factors differed, namely, sex, awareness of the risk of non-surgical cosmetic procedures, and having a health practitioner in the family.

**Table 5:** Total ACSS scores and subscale scores for non-surgical cosmetic procedures according to demographic and other variables M - mean, SD - standard deviation; p < 0.05 (significant)

	Intrapersonal scale (M ± SD: 23.2 ± 8.3)		Social scale (M ± SD: 19.9 ± 10.1)		Consider scale (M ± SD: 20.5 ± 8.3)		Overall score (M ± SD: 63.7 ± 24.5)	
Characteristics	N = 386	p-value^1^	N = 386	p-value^1^	N = 386	p-value^1^	N = 386	p-value^1^
Age (years)		0.003		<0.001		<0.001		<0.001
18–34	22 ± 8		18 ± 10		19 ± 9		60 ± 25	
35–54	25 ± 8		24 ± 9		23 ± 7		72 ± 22	
55 and above	26 ± 6		23 ± 10		22 ± 7		71 ± 21	
Sex		0.017		0.2		<0.001		0.015
Female	24 ± 8		20 ± 10		21 ± 8		66 ± 25	
Male	22 ± 8		19 ± 10		18 ± 8		59 ± 24	
Marital status		<0.001		<0.001		<0.001		<0.001
Single	22 ± 8		17 ± 10		19 ± 8		58 ± 23	
Engaged	26 ± 8		27 ± 9		23 ± 6		77 ± 22	
Married	25 ± 8		22 ± 10		22 ± 9		69 ± 25	
Divorced	29 ± 5		28 ± 7		27 ± 3		84 ± 12	
Widowed	26 ± 5		27 ± 5		25 ± 3		78 ± 9	
Monthly income		0.13		<0.001		0.092		0.009
Less than 5000 Saudi Riyals	25 ± 9		24 ± 10		22 ± 8		71 ± 25	
5000–10,000 Saudi Riyals	24 ± 8		20 ± 10		21 ± 9		64 ± 25	
More than 10,000 Saudi Riyals	22 ± 8		18 ± 10		20 ± 8		61 ± 24	
Do you have a health practitioner in your family?		0.024		0.078		0.058		0.032
No	22 ± 9		19 ± 10		19 ± 9		60 ± 25	
Yes	24 ± 8		21 ± 10		21 ± 8		66 ± 24	
Father’s level of education		0.002		<0.001		<0.001		<0.001
Doesn’t read or write	27 ± 7		27 ± 9		24 ± 7		78 ± 22	
Only he reads and writes	25 ± 9		24 ± 10		22 ± 8		71 ± 27	
Elementary school or middle school	24 ± 8		23 ± 10		22 ± 7		68 ± 23	
High school	24 ± 9		19 ± 11		20 ± 9		63 ± 26	
Bachelor’s degree	24 ± 7		18 ± 9		21 ± 8		63 ± 20	
Diploma	21 ± 8		17 ± 9		18 ± 8		56 ± 24	
Master’s degree or PhD	25 ± 7		24 ± 8		24 ± 7		73 ± 19	
Mother’s level of education		0.034		<0.001		0.031		0.002
Doesn’t read or write	25 ± 9		23 ± 10		22 ± 8		70 ± 26	
Only she reads and writes	24 ± 8		24 ± 9		23 ± 8		72 ± 23	
Elementary school or middle school	23 ± 8		19 ± 11		19 ± 8		61 ± 26	
High school	19 ± 9		15 ± 10		17 ± 10		51 ± 27	
Bachelor’s degree	23 ± 7		20 ± 9		20 ± 8		63 ± 23	
Diploma	23 ± 8		19 ± 10		21 ± 8		62 ± 23	
Master’s degree or PhD	25 ± 7		20 ± 9		22 ± 8		67 ± 18	
Have you ever undergone non-surgical cosmetic procedures?		<0.001		<0.001		<0.001		<0.001
No	23 ± 8		19 ± 10		20 ± 8		62 ± 25	
Yes	29 ± 5		28 ± 6		25 ± 5		82 ± 13	
Are you aware of the risks of non-surgical cosmetic procedures?		0.4		0.2		0.6		0.3
No	24 ± 8		21 ± 10		21 ± 8		66 ± 25	
Yes	23 ± 8		20 ± 10		20 ± 8		63 ± 24	
^1^One-way ANOVA; Welch two-sample t-test								

“I don’t think I need it” was the most commonly cited reason for both surgical and non-surgical cosmetic procedures. Interestingly, religious beliefs were the second most commonly cited reason (Table 6).

**Table 6:** Reasons for non-acceptance of cosmetic procedures in the Jazan population (n = 386)

Reasons for non-acceptance of cosmetic surgery	n (%)
I don’t think I need it	195 (50.52%)
Because it is expensive	73 (18.91%)
I’m afraid of its side effects	81 (20.98%)
I’m afraid of accepting my new look	37 (9.59%)
Social reasons	30 (7.77%)
Religious beliefs	104 (26.94%)
Others	50 (12.95%)
Reasons for non-acceptance of non-surgical cosmetic procedures	
I don’t think I need it	175 (45.34%)
Because it is expensive	79 (20.47%)
I’m afraid of its side effects	83 (21.50%)
I’m afraid of accepting my new look	34 (8.81%)
Social reasons	31 (8.03%)
Religious beliefs	73 (18.91%)
Others	55 (14.25%)

Ages between 35 and 54 years, the lack of employment, employment in the government sector, and previous cosmetic surgery were associated with higher acceptance of cosmetic surgery. Similarly, previous cosmetic procedures, the lack of employment, and employment in the government sector were also associated with higher acceptance of non-surgical cosmetic procedures (Tables 7-8). In contrast, a monthly income higher than 10,000 Saudi Riyals and having a father with an elementary, middle, or high school education, a bachelor’s degree, or a diploma were found to be associated with lower acceptance of cosmetic surgery (Table 5). Further, male sex, divorce, having a father with a diploma, and having a medical practitioner in the family were found to be predictors of lower acceptance of non-surgical cosmetic procedures, but these factors were not associated with acceptance of cosmetic surgery.

**Table 7:** Predictors of acceptance of cosmetic surgery in the Jazan population p < 0.05 (significant) The inclusion of 0 in this confidence interval indicates that there was no valid association between having awareness about the risks of non-surgical cosmetic procedures and acceptance of these procedures.

Characteristics	Beta (95% CI)^1^	p-value
Age (years)		
18–34	—	
35–54	7.9 (1.0 to 15)	0.024
55 and above	7.0 (-8.1 to 22)	0.36
Career status		
Student	—	
Government sector employee	9.3 (1.8 to 17)	0.015
Private sector employee	5.5 (-4.0 to 15)	0.26
Self-employed	-3.9 (-19 to 11)	0.60
Unemployed	13 (5.0 to 21)	0.002
Retired	9.3 (-6.0 to 25)	0.23
Monthly income		
Less than 5000 Saudi Riyals	—	
5000–10,000 Saudi Riyals	-6.8 (-14 to 0.11)	0.054
More than 10,000 Saudi Riyals	-7.2 (-14 to -0.32)	0.040
Father’s level of education		
Doesn’t read or write	—	
Only he reads and writes	-4.4 (-17 to 7.8)	0.48
Elementary school or middle school	-14 (-24 to -2.9)	0.013
High school	-14 (-24 to -3.2)	0.011
Bachelor’s degree	-16 (-27 to -4.6)	0.006
Diploma	-15 (-25 to -5.2)	0.003
Master’s degree or PhD	-1.7 (-14 to 11)	0.79
Are you aware of the risks of plastic surgery?		
No	—	
Yes	-4.7 (-10 to 0.63)	0.084
Have you ever undergone cosmetic surgery?		
No	—	
Yes	21 (13 to 29)	<0.001
^1^CI = confidence interval		

**Table 8:** Predictors of acceptance of non-surgical cosmetic procedures in the Jazan population p < 0.05 (significant) The inclusion of 0 in this confidence interval indicates that there was no valid association between having awareness about the risks of non-surgical cosmetic procedures and acceptance of these procedures.

Characteristics	Beta (95% CI)^1^	p-value
Sex		
Female	—	
Male	-5.1 (-10 to -0.16)	0.043
Marital status		
Single	—	
Engaged	9.0 (-0.81 to 19)	0.072
Married	5.8 (-0.96 to 12)	0.093
Divorced	19 (-10 to -0.16)	0.001
Widowed	5.3 (-12 to 23)	0.55
Father’s level of education		
Doesn’t read or write	—	
Only he reads and writes	-3.4 (-15 to 8.4)	0.57
Elementary school or middle school	-3.2 (-14 to 7.2)	0.55
High school	-7.8 (-18 to 2.3)	0.13
Bachelor’s degree	-9.4 (-20 to 1.5)	0.089
Diploma	-13 (-23 to -3.5)	0.008
Master’s degree or PhD	6.4 (-5.6 to 18)	0.29
Career status		
Student	—	
Government sector employee	9.5 (2.6 to 16)	0.007
Private sector employee	7.4 (-2.0 to 17)	0.12
Self-employed	6.7 (-7.8 to 21)	0.37
Unemployed	8.3 (0.03 to 17)	0.049
Retired	-0.18 (-15 to 14)	0.98
Do you have a health practitioner in your family?		
No	—	
Yes	5.6 (0.73 to 10)	0.024
Have you ever undergone non-surgical cosmetic procedures?		
No	—	
Yes	14 (6.0 to 22)	<0.001
Are you aware of the risks of non-surgical cosmetic procedures?		
No	—	
Yes	-3.7 (-8.8 to 1.4)	0.16
^1^CI = confidence interval		

The main sources of information were social media and the World Wide Web for both cosmetic surgeries (242 and 167; 62.69% and 43.26%, respectively) and non-surgical procedures (223 and 165; 57.77% and 42.75%, respectively). Tummy tuck (71, 18.39%) and rhinoplasty (64, 16.58%) were the most common surgical procedures planned, and laser (134, 34.72%), fillers (107, 27.72%), and Botox (75, 19.43%) were the most common non-surgical procedures (Table 9).

**Table 9:** Sources of information about cosmetic procedures and plans for future procedures in the Jazan population (n = 386)

Source of information about cosmetic surgery	n (%)
Medical references	127 (32.90%)
Family doctor	37 (9.59%)
Social media	242 (62.69%)
Friend	110 (28.50%)
Family	60 (15.54%)
World Wide Web	167 (43.26%)
Other	42 (10.88%)
Future plans for cosmetic surgeries	
Liposuction	38 (9.84%)
Breast enhancement	20 (5.18%)
Hair transplant	40 (10.36%)
Blepharoplasty	34 (8.81%)
Rhinoplasty	64 (16.58%)
Body contouring	25 (6.48%)
Facelift	24 (6.22%)
Septoplasty	25 (6.48%)
Burn repair surgery	20 (5.18%)
Chin augmentation	24 (6.22%)
Scar revision surgery	27 (6.99%)
Tummy tuck	71 (18.39%)
Source of information about non-surgical procedures	
Medical references	126 (32.64%)
Family doctor	44 (11.40%)
Social media	223 (57.77%)
Friend	108 (27.98%)
Family	74 (19.17%)
World wide web	165 (42.75%)
Other	57 (14.77%)
Future plans for non-surgical procedures	
Fillers	107 (27.72%)
Botox	75 (19.43%)
Laser	134 (34.72%)
Facial thread-lift	45 (11.66%)
Tattoo removal	12 (3.11%)

## Discussion

The present study aimed to assess the acceptance of cosmetic surgeries and non-surgical cosmetic procedures in a population from Jazan, Saudi Arabia. The findings are pertinent as there is very little information about these trends in Saudi Arabia, and they could help guide future efforts to increase awareness about the risks of these procedures. Future studies could explore this finding in more detail, perhaps by surveying other populations in Saudi Arabia.

A noteworthy finding of this study was that the overall acceptance of cosmetic surgery among the population was low. While this finding is consistent with a study conducted in Riyadh, Saudi Arabia by Morait et al. [11], the acceptance rate is higher than that reported in studies conducted in the Eastern province of Saudi Arabia [12] and other countries [13,14]. Another important related finding of this study was that non-surgical cosmetic procedures had higher mean ACSS scores than cosmetic surgeries. This may indicate that individuals are more willing to undergo non-invasive, less expensive, and readily available procedures that yield quick and satisfactory results, as reported by a previous Saudi study that explained the difference based on the easy availability of non-surgical cosmetic procedures, their considerably lower price, quicker and reversible results, and lower degree of invasiveness than cosmetic surgeries [15]. Further, the low acceptance of cosmetic surgeries could be related to a lack of access to experienced surgeons and state-of-the-art technologies and a lack of information about these procedures in the region studied.

This could mean that the trends may not reflect the pattern in other regions of Saudi Arabia. Therefore, it is important to survey other regions of Saudi Arabia to understand how this trend plays out across the country in general.

Women exhibited significantly higher scores in the total, interpersonal, and consider subscales for non-surgical cosmetic procedures. At the same time, no significant sex-based differences were observed in the social dimension; this is in alignment with a previous study [11].

In contrast to the findings for non-surgical cosmetic procedures, no significant sex-based differences were observed for acceptance of cosmetic surgery. This observation is in line with that of a Croatian study [16] but contradicts the findings of other studies [10,13,15], which have shown that women tend to have significantly higher scores on the intrapersonal and consider subscales concerning cosmetic surgery. One study [15] has suggested that sex-based differences may result from the greater sociocultural pressure placed on women to achieve physical attractiveness and conform to societal beauty ideals. This applies to the Saudi Arabian context, as this country is known to have various sex-based social and cultural norms. Thus, the influence of social and cultural factors on the acceptance of cosmetic surgery cannot be entirely ruled out, even though the scores for the social subscale did not show significant sex-based differences. In our sample, the male-to-female ratio was skewed in favor of females, but this does not adequately explain our findings. Unfortunately, based on the current data, we are unable to provide a clear explanation as to why there were no sex-based differences in the acceptance of cosmetic surgery.

In the current study, higher acceptance for both cosmetic surgery and non-surgical procedures was found in the older population of this study, that is, those aged above 35 years. This could be attributable to aging-related changes in the skin that are common in older populations. In contrast, Alharethy showed that the most common age range for undergoing cosmetic renovations was 20-40 years, even though individuals older than 40 years of age also expressed a desire for cosmetic enhancements [17]. Moreover, current trends indicate that younger Saudis are more willing to undergo cosmetic procedures [14]. This trend needs to be monitored in the future, as the popularity of these procedures continues to rise. Another influential demographic factor was marital status. In accordance with the findings of several researchers [18,19], unmarried participants in this study expressed a greater desire for cosmetic surgery. This may be attributable to the widespread belief among unmarried individuals that physical beauty is a primary facilitator of marriage, and they probably feel the need to be as physically attractive as possible in order to have a successful marriage. That is, the participants were probably of the belief that enhanced physical appearance would lead to better relationship and marital prospects. It would be interesting to explore the psychosocial factors that play a role in this trend in greater detail in the future.

Evidence shows that self-esteem increases after cosmetic procedures [4]. This may explain why the intrapersonal scale had the highest score across all three subscales for both cosmetic surgeries and procedures. This was followed by the consider subscale for cosmetic surgeries, and the finding indicates that individuals carefully contemplate the potential benefits and risks associated with surgeries. However, the social subscale score was slightly higher than the consider subscale score for non-surgical cosmetic procedures. This indicates that participants consider the impact of social interactions and the experience of fear related to whether their new look will be accepted by themselves or others, and this may serve as a barrier to accepting both surgical and non-surgical cosmetic procedures. These results shed light on the complex psychological factors contributing to individuals’ motivations and decision-making processes in relation to cosmetic surgeries. These findings align with previous research conducted in Saudi Arabia, Serbia, Iran, Italy, South Korea, and Nigeria [11,13-15,20,21] and emphasize the significance of understanding and examining the various dimensions of acceptance of cosmetic operations. Overall, these findings highlight the complex interplay between social pressure to look good based on social media and societal beauty standards, which may positively influence the acceptance of plastic surgery, and stigmatization of cosmetic procedures based on religious or cultural beliefs in certain contexts, which discourages individuals from seeking such procedures and places emphasis on appreciating one’s natural beauty.

An interesting finding that emerged in our study is a statistically significant inverse correlation between the monthly income level and the overall ACSS score. In line with this, only a small percentage of participants indicated that cost was a barrier to opting for cosmetic procedures, both surgical and non-surgical. This implies that the considerable financial implications associated with cosmetic surgeries and procedures did not serve as a deterrent for individuals opting to undergo such treatments. This finding contradicts the findings of previous studies [22]; for example, a research study in Nepal reported that the majority of their participants (62.6%) believed that cosmetic procedures are usually undertaken by individuals of higher social and economic status. The contrasting finding in our sample may be explained by the perception that improving physical appearance may lead to better career and relationship prospects, thereby increasing income and life satisfaction. That is, the study population may hold the belief that improving one’s appearance may be a way to achieve both personal and professional success and may, therefore, not be deterred by the cost of the procedures. 

Employment status was another influential factor in this study. Government sector employees showed a significantly higher ACSS score, which is in alignment with other findings in the literature [22,23]. These findings suggest that individuals with stable employment are more likely to consider and accept cosmetic surgeries, possibly due to their financial stability. However, this appears contradictory to the finding that income level is inversely proportional to acceptance of cosmetic surgeries, which suggests that financial status may not be a deterrent to acceptance of cosmetic surgery. This is a question that needs to be explored further, as the relationships between these factors appear to be complex and multidirectional.

Previous cosmetic procedures also emerged as a predictor of acceptance, in line with a previous study, thus indicating a potential normalizing effect or increased comfort with aesthetic interventions after initial experiences [24]. Another influential factor was elevated paternal education level, which was linked with lower acceptance of cosmetic surgery. Higher education levels of parents could lead to better awareness of the risks of these procedures, but this association was not explored in this study. In contrast to the present findings, previous research found that elevated paternal education level is linked with higher acceptance of cosmetic surgery [25]. It is possible that higher parental education levels are linked with higher financial status, but this is also an association that needs to be explored in the future. As mentioned earlier, the influence of financial status is a topic that needs in-depth study.

The prominent self-perception-driven rationale "I don’t think I need it" was the most frequently cited reason for surgical and non-surgical cosmetic procedures. This finding emphasizes individual motivations and desires as primary factors influencing the decision-making process in cosmetic interventions. Intriguingly, the second most common reason was religious beliefs, which signifies the multifaceted nature of influential factors and points to the need to incorporate cultural and ethical considerations into the decision-making framework.

It is important to note that this study has a few limitations. First, the sample size was relatively small, which may limit the generalizability of the findings to the entire Jazan population. The self-reported nature of the data also poses a limitation, as the possibility of a bias cannot be ruled. Additionally, the study did not explore the underlying reasons and motivations in depth. This could be addressed through future interviews and surveys with open-ended questions in other populations. Another limitation is that co-variants were not evaluated. For example, having previously undergone plastic surgery/procedure was a confounding factor for acceptance of another surgery. Future research could delve deeper into the psychological factors influencing individuals' decisions and explore the impact of cultural and societal norms on the acceptance of cosmetic surgery in Saudi Arabia. More specifically, it might be useful to employ a mixed-methods approach that includes interviews with open-ended questions that will enable a deeper exploration of the interplay of socioeconomic and psychological factors influencing the acceptance of cosmetic surgeries and non-surgical cosmetic surgeries.

## Conclusions

Our findings did not demonstrate any significant sex-based differences in the acceptance of cosmetic surgeries, although women exhibited higher acceptance of cosmetic procedures than men. Moreover, non-surgical cosmetic procedures had higher acceptance scores than cosmetic surgeries. Our findings also showed that sex, marital status, income level, familial influence, and prior experience all played significant roles in shaping acceptance, with age and prior history of procedures having a notable positive impact. An interesting finding of this study was that lower income levels were linked to greater social acceptance of procedures. The most common barriers to accepting surgical and non-surgical cosmetic procedures were the perceived lack of a need for them and religious beliefs. These results emphasize the complex interplay of factors that influence the acceptance of cosmetic interventions. Understanding these acceptance patterns can contribute to the development of targeted interventions and educational programs to promote safe and informed decision-making in relation to cosmetic enhancements in Jazan and other regions of Saudi Arabia.
